# Support for the coverage EMA: Participants can perceive and report discrete auditory event characteristics over 2 h in a simulated EMA scenario

**DOI:** 10.3758/s13428-026-02977-3

**Published:** 2026-03-30

**Authors:** Meynard John L. Toledo, Arthur A. Stone, Olivia L. Pomeroy, Sarah Goldstein

**Affiliations:** 1 Dornsife Center for Self-Report Science & Center for Economic and Social Research, University of Southern California, 635 Downey Way, Los Angeles, CA 90089-3332 USA; 2 Department of Psychology, University of Southern California, Los Angeles, CA USA

**Keywords:** Coverage ecological momentary assessment (cEMA), Recall bias, Self-report ratings, Real-world stimulus recall

## Abstract

Ecological momentary assessment (EMA) minimizes recall bias common in self-report, but variations like the “coverage model” reintroduce short-term recall, raising concerns about accuracy. This study evaluated the fidelity of ratings of characteristics of a discrete auditory event over a 2-h period, simulating coverage EMA, and examined the influence of objective event characteristics (intensity, duration, temporal location) on these ratings. In a remote experiment, participants (*N* = 741) watched a 2-h film containing one embedded thunder sound stimulus. A 2 × 2 × 3 between-subjects design manipulated stimulus intensity (high/low), duration (short/long), and temporal location (start/middle/end). Immediately after, participants completed coverage EMA-style ratings of perceived intensity, duration, and location. Participants' ratings generally reflected the manipulated objective characteristics; the main effects of intensity, duration, and location on their corresponding ratings were significant. Evidence supporting cross-characteristic influences were weak/anecdotal, and effect sizes were small. Approximately 25% of participants failed to accurately report the single event's occurrence; accurate detection was predicted by higher intensity, longer duration, and temporal location (*p* < .01). The results show that coverage EMA reports over a 2-h period can capture basic features of discrete events and demonstrate sensitivity to objective characteristics. They also exhibit certain reporting patterns that suggest potential influences between characteristics, although relatively weak effect sizes. Event detection sensitivity may also vary, particularly for less salient stimuli. These findings highlight the utility of coverage EMA while also emphasizing the need for researchers to consider how these patterns might operate within the context of their specific study goals.

## Introduction

Accurate recall of past events and experiences is fundamental not only for navigating daily life but also for the integrity of scientific research concerning self-reporting. Within numerous disciplines, particularly the behavioral and health sciences, self-report methodologies are indispensable for gathering data on subjective states, behaviors, and exposures to external conditions (Gnanasakthy et al., 2019; Paulhus & Vazire, 2007; Stone & Shiffman, 1994). The usefulness of findings derived from self-reporting hinges upon participants' capacity to remember and articulate their experiences accurately. Biased or unreliable recall of experiences can lead to flawed scientific conclusions and, possibly, misguided interventions.

Recognizing the limitations of retrospective self-reports, researchers have sought methods to capture experiences with minimal retrospection. Ecological momentary assessment (EMA^1^) is a step in this pursuit (Shiffman et al., 2008; Stone & Shiffman, 1994). EMA encompasses a suite of methods designed to collect real-time (“at the moment”) or near-real-time (with a brief period for recall) data on individuals' thoughts, feelings, and behaviors as they go about their everyday lives. By employing frequent, repeated assessments, EMA significantly minimizes the temporal gap between an experience and its report (Shiffman et al., 2008). This approach has been useful for understanding the ebb and flow of states over the course of days in individuals’ natural environments and for characterizing individuals based on their momentary states. For example, research on emotion dynamics employs EMA methods to characterize the trajectories, patterns, and regularities in fluctuations of affect and emotions within people across time (Kuppens, 2015); these findings have been useful in predicting health outcomes such as wellbeing (Houben et al., 2015; Schulz et al., 2021; Xia et al., 2021). Physical activity (PA) researchers have leveraged EMA methods to study processes that occur within an individual (e.g., how a person’s current stress level, affective state, or environmental cues are related to PA behaviors) to better understand how and when to intervene in order to promote healthy behaviors (Dunton, 2017).

Despite its strengths in capturing immediate or near-immediate states, researchers may wish to measure relatively infrequent events or experiences, ones that are likely to occur between scheduled prompts and would likely be “missed” by very brief, momentary assessments. Researchers might also wish to capture summaries of experiences that occur over an interval (e.g., how long a painful episode was experienced). To address these goals, a variation of the momentary EMA method has been developed that asks participants to report on events or experiences in a relatively short timeframe (e.g., since the last prompt, within the last 2 h) (May et al., 2018; Stone et al., 2023). This is known as the “coverage” approach to EMA or cEMA (May et al., 2018; Stone et al., 2023). The technique reduces the duration of the recall window compared to traditional surveys, which might ask about the last week or month, yet it introduces an element of retrospection to EMA. Consequently, the accuracy of cEMA is a concern and should be empirically verified.

This concern is sensible. Although one might initially assume that recall over the brief intervals used in cEMA approaches (like the last hour or two) would be fairly accurate, research in the field of pain experiences suggests otherwise (Ariely, 1998; Bellur & Sundar, 2014; Kahneman et al., 1993). Biases due to cognitive heuristics have been shown to influence recall over short periods (under an hour), which may be viewed as a concern for the fidelity of data collected with cEMA. Studies by Kahneman and colleagues provided striking examples: participants preferred repeating a 90-s cold-water immersion ending with slightly lessened pain over a shorter, 60-s immersion ending at peak pain, effectively choosing more total pain because the last few seconds of that condition generated less pain (Kahneman et al., 1993). This “peak-end rule,” combined with “duration neglect,” was also observed in patients recalling painful medical procedures like colonoscopy or lithotripsy where participants’ memory of their pain for the entire procedure correlated strongly with the highest level of pain in the procedure (“peak”) and pain levels in the final moments of the procedure (“end”). Moreover, the procedure’s duration (which varied from minutes to over an hour) was largely unrelated to the reporting of pain for the whole procedure (Redelmeier et al., 2003). Additional compelling evidence is found in research from Ariely and colleagues (Ariely, 1998) that documented similar memory distortions over even shorter durations (i.e., immediately after experiencing the stimulus). Studying pain induced by heat or pressure and then recalled, Ariely showed that retrospective evaluations were biased by the pattern of intensity change (e.g., increasing vs. decreasing), the rate of change, and the final intensity level (Ariely, 1998). Both Kahneman’s and Ariely’s work show that recall over a period of minutes can be distorted by heuristics and memory processes (e.g., simple forgetting), implying that recall over “the last hour or two” interval used by cEMA could be susceptible to bias.

On the other hand, although the potential for recall biases from heuristics has been shown over short periods, other research suggests the practical impact on the accuracy of recall in daily life contexts might be limited. A study by Schneider and colleagues (Schneider et al., 2011) examined daily recall of pain and fatigue in rheumatology patients and found that while peak and end pain influenced end-of-day recall, they only account for 2–3% of additional variance beyond the prediction of recall from the average of momentary reports. These small effects observed in the field highlight the uncertainty about how reliably people recall experiences over the typical intervals used in cEMA. This underscores the need for investigation designed to assess recall fidelity of the cEMA methodology to evaluate its accuracy.

The present study was designed to investigate the accuracy of 2-h recall of a brief, auditory stimulus. A strength of the experimental design was that the auditory stimuli to be recalled were designed so that three stimulus characteristics were varied and then assessed at the end of the procedure. In short, participants watched a 2-h nature video depicting ocean wildlife. Embedded within this continuous experience was a short burst of thunderstorm sounds. This stimulus was selected for several reasons. First, thunder represents a relatively unambiguous perceptual event whose basic characteristics (like perceived loudness and length) participants could reasonably be expected to recall, especially in contrast to neutral underwater scenes. Second, we could manipulate the objective characteristics of the auditory event—its intensity (i.e., volume), duration, and temporal location. Accordingly, participants were randomly assigned to one of 12 conditions representing a unique combination of intensity (two levels), duration (two levels), and temporal location (three levels). After watching the video, participants answered a series of questions about the characteristics of the thunder in a manner analogous to questions used in cEMA.

Our overall aim was to contribute evidence regarding the utility of cEMA by evaluating the accuracy of participants' short-term recall ratings for a discrete auditory event. The accuracy of 2-h recall was assessed by determining if the cEMA ratings reflected the experimentally based stimuli characteristics. We advance two hypotheses:

### Hypothesis 1: cEMA ratings will be associated with corresponding stimuli characteristics

 Each of the manipulated stimulus characteristics (intensity, duration, temporal location) will be significantly associated with its corresponding cEMA rating. That is, the ratings of intensity will be higher for the groups exposed to higher-intensity stimuli compared with the intensity ratings of those exposed to lower-intensity stimuli and so on for duration and temporal location. This hypothesis refers directly to the question of the accuracy of recall over the 2-h reporting period.

### Hypothesis 2: A given cEMA rating of the stimulus will not be associated with stimuli characteristics other than the one being rated

 Each of the stimulus characteristics will not be associated with cEMA ratings of the other stimulus characteristics; for example, groups exposed to the low- and high-intensity stimuli will not show evidence of rating differences in duration or the temporal location cEMA ratings. This refers to the question of whether the construct being rated is influenced only by the stimulus characteristics that were intended to affect it. For example, if differences in intensity influenced the duration cEMA ratings (where duration ratings were on average the same for both groups of intensity stimuli), then one would question the meaning of duration ratings.

### Exploratory analyses

These analyses were generated at the early stage of data analysis when we realized that some participants did not report hearing only one instance of thunder: most of these instances involved participants reporting that they did not hear any thunder and other participants reported hearing more than one instance of thunder. The analyses compared the demographic and stimuli characteristics to which participants were exposed in order to understand the reason for these “errors” and how they might modify the conclusions of the two primary hypotheses.

Our hope was that findings from this study would inform future strategies for designing cEMA protocols and contribute to ongoing efforts to improve the quality of self-report methods for capturing the complexities of everyday experience.

## Methods

### Participant sample

Participants were recruited from Prolific, an online research platform with over 200,000 active participants. Any participant whose self-reported demographic and health information on Prolific matched the study’s pre-screening criteria could view and access the study. To meet the pre-screening criteria, participants had to have completed at least 25 previous submissions with a minimum approval rating of 99% on Prolific; additionally, participants were eligible if they (1) were at least 18 years old; (2) were able to read and comprehend the English language fluently; (3) were located in the United States; (4) had normal or corrected-to-normal vision; (5) did not have any hearing loss or hearing difficulties; (6) did not have attention deficit disorder (ADD) or attention deficit hyperactivity disorder (ADHD); and (7) did not have any cognitive impairment or dementia. Participants also had to have access to a personal laptop or desktop computer and reliable wireless internet to participate. There were 1,183 Prolific participants who were assessed for eligibility in our screening survey, and 1,139 met the eligibility criteria. A total of 1,125 participants consented to participate in the study, and 1,000 participants completed the full study protocol.

### Study design

#### Study procedures

The study protocol was executed online and was designed to last a total of 2 h and 30 min. Participants were instructed to complete the study in a quiet, private space without interruption. After providing consent, participants were asked to review detailed instructions about the study protocol and answer comprehension questions to confirm their understanding. Participants had to answer all comprehension questions correctly within two attempts to be able to proceed to the demographic questions.

Participants then completed the audio adjustment task, which guided them through setting their computer volume to a comfortable level in preparation for the 2-h video activity. This process aimed to reduce the risk of participants adjusting their volume during the 2-h video activity and create a more standardized participant experience of the thunder stimulus. All participants had the option of using headphones to complete the study protocol, and if they chose to use them, they were instructed to wear them while completing the audio adjustment task and not remove them at any point during the 2-h video activity. Participants first listened to an audio clip representing the 2-h video activity’s typical volume before being instructed to adjust their computer volume to a comfortable level. Next, they listened to a second audio clip representing the loudest volume they would hear during the 2-h video activity, and if this clip was intolerable, they were instructed to return to the first audio clip and complete the audio adjustment process again. After completing this process, participants were asked to not adjust their computer volume during the rest of the study. The loudness of the typical volume audio clip was determined by approximating the average decibels across various points throughout the 2-h video activity. The loudness of the “loudest” volume audio clip corresponded to the high-intensity thunder stimulus. This clip was set approximately 13 decibels higher than the typical volume audio clip. Consequently, the low-intensity thunder stimulus was set 10 decibels lower than the high-intensity stimulus, resulting in a level approximately 3 decibels higher than the average background volume, a difference generally considered the smallest change in sound level noticeable to the human ear (Federal Highway Administration, 2018). Participants were instructed not to adjust their volume or remove their headphones (if applicable) at any point during the 2-h video activity.

Participants were randomized to one of 12 study conditions (see Table 1) that determined the duration, intensity, and temporal location of the thunder stimulus, and they were redirected to begin the 2-h video activity. The video activity involved watching a 2-h series of documentary films about ocean life with the thunder stimulus inserted at either the 30-min (start), 60-min (middle), or 90-min (end) mark depending on their condition. The thunder stimulus lasted for either 15 s (short duration) or 45 s (long duration). To ensure participants paid attention throughout the 2-h video activity, participants were asked to click on the 12 red target symbols that periodically appeared on the screen. Afterwards, participants answered a post-study questionnaire about their recollection of the thunder stimulus and their experience watching the 2-h video activity over the prior 2 h. Participants who answered these final questions were asked to complete their submission on Prolific; once their successful study completion was confirmed, their submission was approved, and they were paid $50 through Prolific.

**Table 1:** Full factorial design of the study (2 × 2 × 3)

Group#	Stimulus factors			No. randomized	No. analyzed	% Lost
	Intensity	Duration	Location			
1	High	Long	Start	88	73	17.0
2	Low	Long	Start	87	76	12.6
3	High	Short	Start	85	72	15.3
4	Low	Short	Start	88	48	45.5
5	High	Long	End	86	68	20.9
6	Low	Long	End	86	57	33.7
7	High	Short	End	88	70	20.5
8	Low	Short	End	86	33	61.6
9	High	Long	Middle	86	68	20.9
10	Low	Long	Middle	87	58	33.3
11	High	Short	Middle	87	69	20.7
12	Low	Short	Middle	88	49	55.7

### Materials

#### 2-h video activity

The 2-h video activity was a series of three documentaries about ocean life that lasted for a total of 2 h. As the content of the films was not relevant to the study purpose, the study team considered documentaries that (1) were likely to be engaging to participants, (2) did not already have sounds in them that closely resembled the thunder stimulus, (3) were available for download on YouTube, and (4) had a Creative Commons license which allowed for reuse and editing of the videos. The study team ultimately chose three documentaries on ocean life to provide a consistent theme for the 2-h video activity and because these films could be edited together on iMovie for a total duration of exactly 2 h. Scenes that were overly violent, gory, or distressing were removed from the films to avoid unnecessarily upsetting participants. To measure participant engagement, attention checks were added throughout the 2-h video activity. Twelve red target symbols were displayed in random locations on the computer screen, and participants were asked to click on them as quickly as possible within 60 s to demonstrate they were paying attention.

The 2-h video activity was administered to participants on NubiS, a data collection software tool developed by the Understanding America Study team at the University of Southern California. Participants were automatically redirected between Qualtrics and NubiS in order to facilitate a seamless participant experience.

#### Audio stimulus

A thunder sound was selected as the audio stimulus for the study protocol because it is a universally recognizable sound, which made it a more accessible stimulus for participants to recall when asked about “the thunder” they heard during the 2-h video activity. Further, thunder would stand out as unnatural in the context of the underwater ocean documentaries used in the 2-h video activity, making it a novel stimulus that was clearly intended to be separate from the documentaries. Additionally, the fairly uniform sound of the thunder clip allowed us to manipulate the stimulus in the desired ways for this study, which was essential to examine the research questions. For example, the continuous pattern of the thunder clip made it easy to shorten or lengthen to a 15- or 45-s duration, and the loudness of the thunder could be amplified or reduced to a higher or lower intensity. Finally, the temporal location of the thunder could also be modified to the start (i.e., 30 min from the start), middle (i.e., 60 min from the start), or end (i.e., 90 min from the start) of the 2-h video activity.

### Measures

#### Demographics

Participants were asked demographic questions about their age, gender, ethnicity, race, education, marital status, employment status, and income.

#### Post-study questionnaire

Upon completion of the 2-h video activity, participants were asked questions about their recollection of the thunder stimulus that simulated a cEMA model. To determine if participants recalled hearing the thunder stimulus, they were asked, “Do you remember hearing anything that sounded similar to thunder, gunshots, explosions, or rumbling at any point during the films?” The question included “thunder, gunshots, explosions, or rumbling” because these were the most commonly used descriptions of the stimulus by participants during the pilot testing of the study protocol.

Participants were also asked three other questions about events that did not occur (e.g., “Do you remember seeing any animated or cartoon sea creatures during the films?”) to reduce the risk of suggestion bias unintentionally priming participants to report hearing the thunder. The presentation order of the four questions was randomized for all participants, and they had an opportunity to review and adjust their answers if desired before proceeding with the questionnaire. Regardless of their answers to the stimulus detection questions, all participants were then informed that a thunder stimulus had been inserted into the 2-h video activity, and they were asked to think about any thunder they might have heard over the entire 2-h period while answering the following questions.

### Outcomes

To facilitate clear discussion, this paper will use the term “factor” to refer to the experimental manipulations of the sound stimulus (intensity, duration, and temporal location). The term “rating” will refer to the participants' responses to the EMA questions about these sound characteristics.

#### Sound intensity ratings

Participants were asked to rate the thunder’s intensity by answering the question, “How would you describe the overall loudness of the thunder that you experienced over the last 2 h?” on a scale from 0 to 100, where 0 represented “no sound at all” and 100 represented “the loudest possible sound.”

#### Duration ratings

Participants were asked to estimate the duration of the thunder by answering the question, “How many seconds in total would you estimate that the thunder lasted?” by entering a number between 0 and 7,200. If the participant entered a number less than 10, they were asked to confirm that they had provided their answer using the correct units (i.e., seconds instead of minutes) and were given the opportunity to adjust their answer if needed. This confirmation was motivated by observations of pilot study participants incorrectly using minutes instead of seconds.

#### Temporal location ratings

Participants were asked about the temporal location of the thunder within the 2-h time period with the question, “How many minutes before the end of the 2-Hour Video Activity would you estimate that you experienced the thunder?” Participants provided their answer by dragging a slider along a scale from 0 to 120, where 0 was labeled “End of the 2-Hour Video Activity,” 60 was labeled “Midpoint of the 2-Hour Video Activity,” and 120 was labeled “Start of the 2-Hour Video Activity.” Due to the tendency of pilot study participants to misinterpret this question and report their answer relative to the start of the 2-h video activity rather than the end, participants were asked to review their response and were given the opportunity to adjust their answer if they had initially misinterpreted the question. Furthermore, participants who answered there was more than one separate period of thunder were asked about the temporal location for each period of thunder they reported.

#### Frequency ratings

Participants were asked to recall the frequency of the thunder by answering the question, “How many separate periods of thunder did you experience over the last 2 h?” using a number between 0 and 3. The correct answer to this question was one period of thunder. Participants who answered 0 were reminded that they should only answer 0 if they heard no thunder at all over the 2-h period and had the opportunity to adjust their answer if they had initially misinterpreted the question.

### Statistical analysis plan

The main goal of the study was to determine if participants could accurately recall the three manipulated characteristics of a discrete auditory stimulus using the cEMA questions that immediately followed the 2-h video activity. The study employed a between-subjects 2 (Intensity: high, low) × 2 (Duration: long, short) × 3 (Temporal Location: start, middle, end) factorial design, and participants were randomly assigned to one of the 12 conditions. All analyses were conducted using R version 4.3.1. Alpha was set to 0.05 for all statistical tests.

Descriptive statistics were used to describe the sample and summarize the outcome variables. Data visualization methods including histograms, box plots, and scatterplots were employed to inspect the distributions, assess normality, and identify potential outliers. Values exceeding ± 3 standard deviations (SD) from the mean were considered outliers. All outliers were Winsorized (replaced with the next lowest non-outlying value).

To test hypotheses, we conducted three separate three-way analyses of variance (ANOVAs), one for each of the cEMA rating outcomes (intensity rating, duration rating, and temporal location rating). For each ANOVA, the independent variables were the manipulated stimulus characteristics: stimulus intensity (low vs. high), stimulus duration (short vs. long), and stimulus temporal location (start vs. middle vs. late). We report main effects of each characteristic, all two-way interactions, and the three-way interaction. Significant effects were followed up with post hoc pairwise comparisons using Tukey's honestly significant difference (HSD) correction. Effect sizes are reported as partial eta squared (${\upeta }_{p}^{2}$​) for ANOVA effects and Cohen's *d* for pairwise comparisons.

To test Hypothesis 1, we evaluated the main effect of each stimulus characteristic on its corresponding cEMA rating within the ANOVAs (e.g., the main effect of the intensity condition on the intensity rating). In addition, to more directly gauge the accuracy of cEMA recall, we employed specific methods tailored to each characteristic. Because the objective stimulus properties (decibels) and the subjective cEMA ratings were not on the same scale, a direct error calculation was not appropriate. We therefore evaluated how well the subjective ratings discriminated between the low- and high-intensity stimulus groups by examining the non-overlap between their rating distributions. Rom's non-overlap (NOL) index (Rom & Hwang, 1996) was calculated, which indicates the percentage of non-overlap between two distributions, and Cohen's U3​, which measures the percentage of ratings in one group that fall below the median rating of the other group. Values for NOL greater than 0% and U3 greater than 50% suggest discrimination between groups, with higher values reflecting stronger separation. For the duration and temporal location characteristics, the objective and subjective measures were on comparable scales (seconds and minutes, respectively). We therefore calculated the absolute error for each participant by taking the absolute difference between their rating and the true value. We then calculated the percentage of participants whose ratings fell within specific accuracy thresholds: ± 5, ± 15, and ± 30 s for duration, and ± 5, ± 15, and ± 30 min for temporal location. These percentages provide a direct measure of recall accuracy. To aid with interpretation of these results, we also present the distribution of these ratings.

To test Hypothesis 2, we first examined the non-corresponding main effects and interactions from the ANOVAs. For example, to assess the specificity of the duration and temporal location ratings (which were expected to be influenced by the duration and location, respectively), the main effect of the stimulus intensity factor on both of these ratings was evaluated. While nonsignificant *p* values suggest a lack of influence, newer statistical techniques allow for a more convincing argument for a null effect. Therefore, to more rigorously test for the absence of effects where they were not expected, we supplemented our ANOVAs with Bayesian analysis of effects using JASP (JASP Team, 2025). Unlike standard testing, Bayesian methods quantify the strength of evidence in favor of the null hypothesis (i.e., in our case, that an experimental factor had no effect on a non-corresponding rating). We calculated exclusion Bayes factors (BF_01_), which represent the ratio of evidence for a model that excludes a specific factor versus a model that includes it (Kelter, 2021). Consistent with the literature, a BF_01_ > 3 was considered moderate evidence for the absence of an effect, and a BF_01_ > 10 was considered strong evidence (Quintana & Williams, 2018).

Finally, recognizing that some participants did not report a single discrete thunder event (reporting zero or multiple events), exploratory analyses were conducted to identify potential factors associated with these reports. This analysis employed two distinct sets of logistic regression models. The first set compared participants reporting no thunder events (*n* = 133) to those reporting one event correctly (*n* = 741). The second set compared participants reporting multiple thunder events (*n* = 111) to the correct reporters. In these models, potential predictors examined individually included pre- randomization demographic variables (age, gender, education level, income, ethnicity, race) and the manipulated stimulus characteristics (intensity, duration, temporal location). Odds ratios (OR) and standard errors (SE) were calculated for each predictor. Detailed results of this exploratory analysis are presented in Supplemental Tables A1 and B.

## Results

**Fig. 1:**
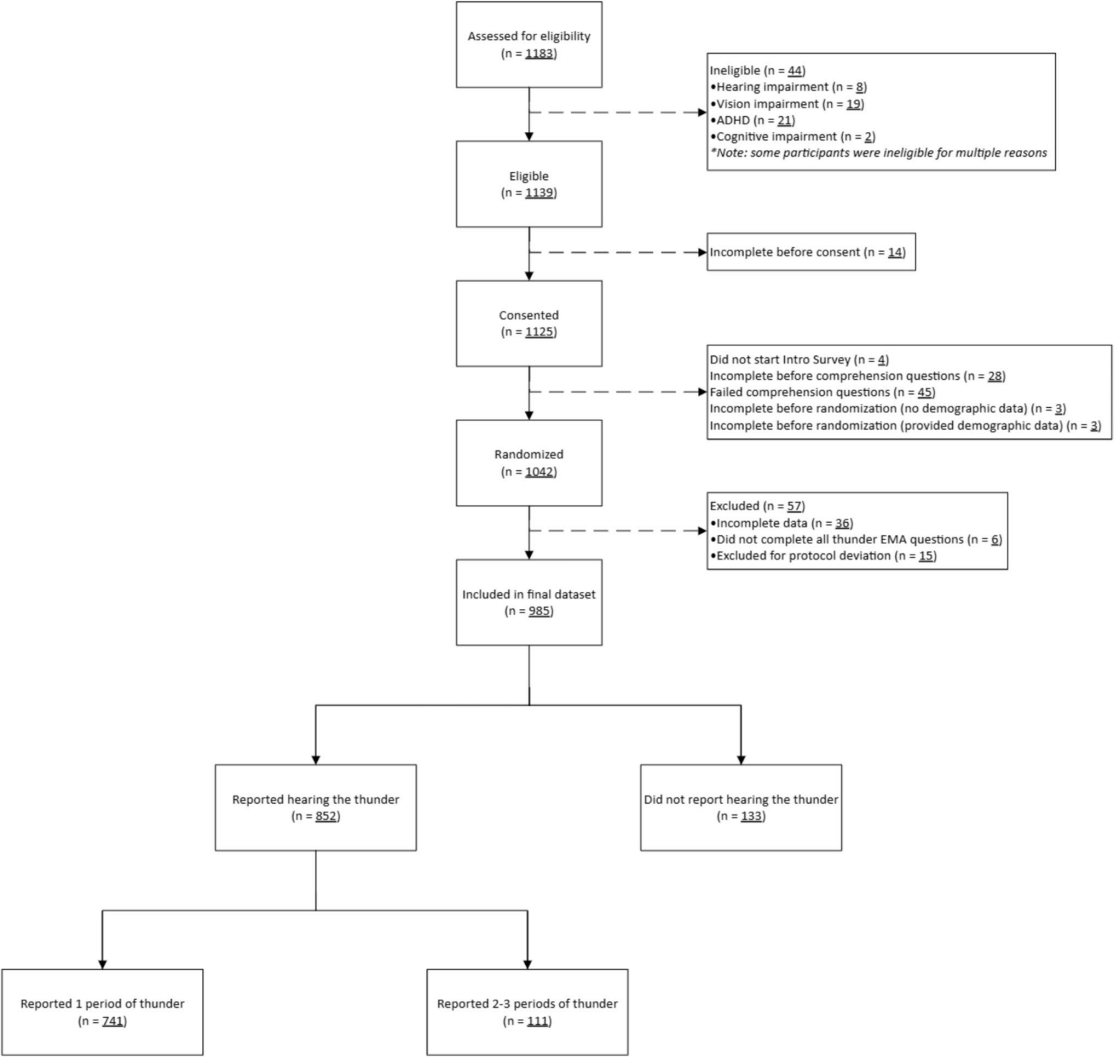
Study flow diagram

The overall recruitment flow is summarized in the consort diagram (Fig. 1). Of the 1,183 people who initially expressed interest in the study, 1,125 provided informed consent. After excluding the 38 participants who did not finish the introductory survey and the 45 who failed comprehension checks, 1,042 participants were randomized to the 12 cells of the factorial design. Of those randomized, 1,000 completed the EMA questions about the thunder stimulus. After excluding 57 participants due to incomplete data (*n* = 42) and protocol deviation (*n* = 15), the final dataset comprised 985 participants, and 852 reported hearing the thunder stimulus. Of these 852 participants, 741 correctly reported a single instance of thunder, while 111 reported two or three instances of thunder. Because the hypotheses are predicated on the participants being able to perceive and report experiencing a single thunder stimulus, the succeeding analyses evaluating the hypotheses presented in this study focused on the 741 participants who reported a single instance of thunder. While it could be argued that participants who reported hearing more than one instance of thunder (*n* = 111) may have reported the correct thunder stimulus in addition to other instances of thunder that did not occur, it was unclear how this misperception might influence ratings. Thus, they were excluded from the analysis of the first two hypotheses. Table 2 summarizes the demographic characteristics of the study participants. The overall study sample who completed the study was, on average, 36.1 years old (*SD* = 11.6). The sample had more women (56.5%), identified as White (58.7%), and was non-Hispanic in ethnicity (87.5%). The sample was roughly evenly split between participants reporting less than college-level education (48.8%) and those with a college education or above (50.9%), and the median income was $60,000. Table 3 summarizes the participant ratings of the stimulus events.

**Table 2:** Demographic characteristics of the study sample

		Misperceived the thunder stimulus		
	Reported one thunder (*n* = 741)	Reported no thunder (*n* = 133)	Reported more than one thunder (*n* = 111)	Total (*N* = 985)
Age	36.4 (11.6)	35.4 (11.7)	34.9 (11.5)	36.1 (11.6)
Gender				
Male	43.5 (322)	37.6 (50)	36 (40)	41.8 (412)
Female	54.9 (407)	61.7 (82)	60.4 (67)	56.5 (556)
Other	1.6 (12)	0.8 (1)	0.9 (1)	1.4 (14)
Race				
White	63.3 (469)	45.1 (60)	44.1 (49)	58.7 (578)
Black	17.7 (131)	32.3 (43)	36 (40)	21.7 (214)
Asian	8 (59)	9 (12)	6.3 (7)	7.9 (78)
American Indian/Native Hawaiian	6.9 (51)	9.8 (13)	8.1 (9)	7.4 (73)
Multiracial	0.9 (7)	0 (0)	1.8 (2)	0.9 (9)
Other	2.3 (17)	1.5 (2)	1.8 (2)	2.1 (21)
Ethnicity	0.9 (7)	2.3 (3)	1.8 (2)	1.2 (12)
Hispanic	12.6 (93)	12.8 (17)	8.1 (9)	12.1 (119)
Non-Hispanic	87.2 (646)	87.2 (116)	90.1 (100)	87.5 (862)
Education				
Less than college level	52.9 (392)	47.4 (63)	41.4 (46)	48.8 (481)
College and above	46.8 (347)	51.9 (69)	58.6 (65)	50.9 (501)
Income				
Less than $60,000	48.7 (361)	40.6 (54)	38.7 (43)	46.5 (458)
$60,000 and above	47.8 (354)	55.6 (74)	57.7 (64)	50 (492)

For continuous variables, summary statistics are reported as mean (*SD*), while categorical variables are reported as percentage (*N*). Income categories were median split. Missing demographic data were observed across several categories: three participants did not report their gender, 12 did not report their race, four did not report their ethnicity, three did not report their education level, and 35 did not report their income level

**Table 3:** Descriptive statistics of stimulus event by condition

	Intensity rating	Duration rating (s)	Location rating (min)
	Mean (*SD*)	Mean (*SD*)	Mean (*SD*)
Intensity factor			
Low	57 (54.6–59.3)	48.4 (44.3–52.6)	58.7 (56.4–61.1)
High	71.7 (69.8–73.7)	42.5 (39–46)	59.2 (57.3–61.2)
Duration factor			
Low	63 (60.7–65.2)	25.6 (21.6–29.7)	60.1 (57.8–62.4)
High	65.7 (63.7–67.7)	65.3 (61.6–68.9)	57.9 (55.8–59.9)
Location factor			
End	66.7 (63.9–69.5)	42.2 (37.2–47.2)	79.5 (76.7–82.3)
Middle	64.9 (62.3–67.5)	44.1 (39.4–48.7)	56.5 (53.9–59.1)
Start	61.5 (59–64)	50.1 (45.6–54.6)	41 (38.5–43.5)

### Hypothesis 1: cEMA ratings will be associated with corresponding stimuli characteristics

**Fig. 2:**
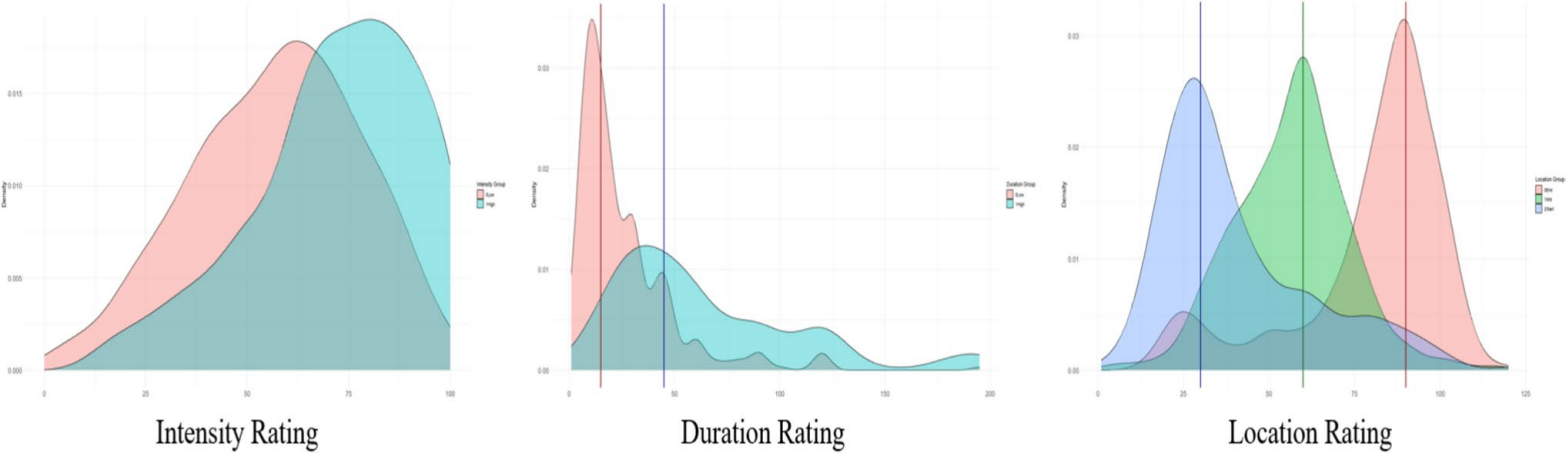
Density plots of the ratings of the thunder stimuli characteristics

The stimulus intensity factor had a significant effect on intensity ratings, *F*(1, 729) = 91.5, *p* <.0001, ${\upeta }_{p}^{2}$= 0.11 (Table 4), confirming the hypothesized expectation. Participants assigned to the high-intensity condition reported significantly higher intensity ratings (mean [95% CI] = 57 [54.6–59.3]) compared to those assigned to the low-intensity group (71.7 [69.8–73.7]), an average difference of ~ 15. Rom’s non-overlap index is 27.7%, indicating that a notable portion of the high-intensity group scored above the low-intensity group (see Fig. 2). Furthermore, a large percentage (Cohen's U3 of 76.1%) of the high-intensity group ratings exceeded the low-intensity group's mean ratings. These analyses collectively illustrate a clear separation between the two intensity conditions, despite considerable distributional overlap, highlighting the robust influence of stimulus intensity on the ratings.

**Table 4:** Three-way ANOVA results for all cEMA ratings by stimulus characteristics

Intensity rating									
IV	SS	*df*		MS	*F*		*p*		${\upeta }_{p}^{2}$
Main effects									
Intensity factor	**38,579**	**1**		**38,579**	**91.50**		** <.0001**		**.11**
Duration factor	1,128	1		1,128	2.68		.102		.004
Location factor	**2,969**	**2**		**1,484**	**3.52**		**.030**		**.01**
Interaction									
Intensity × Duration	1,143	1		1,143	2.71		.100		.004
Intensity × Location	2,037	2		1,019	2.42		.090		.007
Duration × Location	118	2		59	0.14		.869		.0004
Intensity × Duration × Location	181	2		90	0.21		.807		.001
Within-group (error)	307,362	729		422					
Duration rating									
IV	SS	*df*		MS	*F*		*p*		${\upeta }_{p}^{2}$
Main effects									
Intensity factor	**19,733**	**1**		**19,733**	**14.61**		**.0001**		**.02**
Duration factor	**269,417**	**1**		**26,9417**	**199.45**		** <.0001**		**.17**
Location factor	**8,348**	**2**		**4,174**	**3.09**		**.046**		**.006**
Interaction									
Intensity × Duration	5,096	1		5,096	3.77		.052		.004
Intensity × Location	2,867	2		1,433	1.06		.347		.003
Duration × Location	1,664	2		832	0.62		.540		.002
Intensity × Duration × Location	1,870	2		935	0.69		.501		.003
Within-group (error)	1,409,709	983,401		728	1,351				
Location rating									
IV	SS		*df*	MS		*F*		*p*	${\upeta }_{p}^{2}$
Main effects									
Intensity factor	1,000		1	1,000		2.38		.124	.003
Duration factor	1,066		1	1,066		2.53		.11	.003
Location factor	**187,924**		**2**	**93,962**		**223.31**		** <.0001**	**.38**
Interaction									
Intensity × Duration	21		1	21		0.05		.823	0.000
Intensity × Location	690		2	345		0.82		.441	.002
Duration × Location	780		2	390		0.93		.396	.003
Intensity × Duration × Location	1,259		2	630		1.50		.225	.004
Within-group (error)	306,323		728	421

Turning to the stimulus duration factor, it had a highly significant main effect on duration ratings, *F*(1, 728) = 199.45, *p* <.0001, ${\upeta }_{p}^{2}$ = 0.17 (Table 4), also confirming the hypothesis. Participants exposed to the long-duration stimulus reported significantly longer duration ratings (65.3 [61.6–68.9] s) than those exposed to the short-duration stimulus (25.6 [21.6–29.7] s), a difference of ~ 40 s. Examining the accuracy of the duration ratings (directly compared to true duration value; see Table 5), participants in the low-duration group (*n* = 341) overestimated the duration by an average of 11.0 s (*SD* = 24.4, mean absolute deviation [*MAD*] = 15.7). A substantial proportion of participants were relatively accurate: 45.2% of duration ratings within 5 s of the true value, 76.5% within 15 s, and 89.1% within 30 s. Participants in the high-duration group (*n* = 400) also overestimated the duration, with a mean difference of 20.1 s (*SD* = 45.1, *MAD* = 33.7). Accuracy was lower in this condition compared to the low-duration group: 19.0% of participants were within 5 s of the true value, 49.9% within 15 s, and 65.7% within 30 s. Across both duration conditions (*n* = 741), the mean difference was 15.9 s (*SD* = 37.3, *MAD* = 25.4), indicating a general tendency towards overestimation. Overall, 31.1% of participants were accurate within 5 s.

**Table 5:** Accuracy of duration and location ratings in cEMA

Duration	*N*	Mean difference	*SD*	*MAD*	*SD*	% Within 5 s	% Within 15 s	% Within 30 s
Low	341	11.0	24.4	15.7	21.6	45.2	76.5	89.1
High	400	20.1	45.1	33.7	36.0	19.0	49.9	65.7
Total	741	15.9	37.3	25.4	31.5	31.1	62.2	76.5
Location	*N*	Mean difference	*SD*	*MAD*	*SD*	% Within 5 min	% Within 15 min	% Within 30 min
End	228	− 10.3	22.0	15.4	18.9	38.2	74.1	84.2
Middle	244	− 3.4	16.5	12.5	11.2	33.7	70.0	93.4
Start	269	10.6	22.4	16.8	18.1	33.8	65.8	82.9
Total	741	− 0.4	22.3	15.0	16.5	35.1	69.7	86.8

Also confirming the first hypothesis, the temporal location factor had a robust main effect on location ratings, *F*(2, 728) = 223.31, *p* <.0001, ${\upeta }_{p}^{2}$= 0.38 (Table 4). Post hoc analyses with Tukey's HSD correction revealed significant differences between all pairs of locations: Middle versus End (diff =  − 23.08 min, 95% CI [− 27.52, − 18.63], *p* <.0001), Start versus End (diff =  − 39.05 min, 95% CI [− 43.39, − 34.71], *p* <.0001), and Start versus Middle (diff =  − 15.97 min, 95% CI [− 20.24, − 11.71], *p* <.0001). Regarding the accuracy of the location ratings (see Table 5), participants who experienced the stimulus at the end of the film (*n* = 228) reported its location an average of 10.3 (*SD* = 22.0, *MAD* = 15.4) min earlier in the video than the temporal location specified in the study design. Accuracy for this condition (end location) was moderate: 38.2% of participants were within 5 min of the true location, 74.1% within 15 min, and 84.2% within 30 min. Participants in the middle location condition (*n* = 244) had the highest accuracy, with a mean difference of only − 3.4 (*SD* = 16.5, *MAD* = 12.5) min earlier than the design specification. A substantial proportion of participants in this condition (middle location) reported locations close to the true value: 33.7% within 5 min, 70.0% within 15 min, and 93.4% within 30 min. Lastly, participants who experienced the thunder event at the start of the film (*n* = 269) reported its location an average of 10.6 (*SD* = 22.4, *MAD* = 16.8) min later in the film than the design-specified location: 33.8% were within 5 min, 65.8% within 15 min, and 82.9% within 30 min in this condition (start location). Overall, across all location conditions (*n* = 741), the mean difference was 0.4 (*SD* = 22.3, *MAD* = 15.0) min earlier. Overall, 35.1% of responses were within 5 min of the actual location, 69.7% within 15 min, and 86.8% within 30 min.

### Hypothesis 2: cEMA ratings will not be associated with stimuli characteristics other than the one being rated

This hypothesis concerns the possible effect of the experimental factors on ratings that should not be affected by the factors. Contrary to our prediction of no effects on other ratings, the stimulus intensity factor had a significant main effect on duration ratings, *F*(1, 728) = 14.61, *p* =.0001), where participants exposed to higher-intensity stimuli had shorter duration ratings, although the effect size was small (${\upeta }_{p}^{2}$ = 0.02; see Table 4). Complementing this finding, the Bayesian analysis (see Table 6) indicated that the data only slightly favored the model including the effect over the null hypothesis without the effect (BF_01_ = 0.573); by convention, this is considered weak support for the effect. On the other hand, the stimulus intensity factor had no main effect on temporal location ratings, *F*(1, 728) = 2.38, *p* =.124, η^2^ = 0.003, indicating that judgments of temporal location were not influenced by stimulus intensity. This is consistent with the Bayesian analysis providing strong evidence for the null hypothesis (BF_01_ = 11.21), indicating the data were over 11 times more likely to occur under a model where intensity had no influence on location judgments.

**Table 6:** Bayesian analysis of effect

	Intensity ratings			Duration ratings			Location ratings		
	P(inc|Data)	P(excl|Data)	BF_01_	P(inc|Data)	P(excl|Data)	BF_01_	P(inc|Data)	P(excl|Data)	BF_01_
Intensity	.825	<.001	<.001	.49	.28	0.57	.08	.91	11.21
Duration	.20	.71	3.46	.76	<.001	<.001	.23	.75	3.18
Location	.28	.62	2.21	.131	.85	6.49	.98	<.001	<.001
Intensity × Location	.09	.28	3.02	.01	.09	12.32	.01	.08	16.01
Intensity × Duration	.09	.21	2.36	.23	.50	2.11	.003	.02	7.90
Duration × Location	.004	.11	28.17	.01	.14	17.64	.02	.24	13.85
Intensity × Duration × Location	<.001	<.001	13.75	<.001	<.001	6.11	<.001	<.001	4.97

The stimulus duration factor had neither a significant main effect on intensity ratings, *F*(1, 729) = 2.68, *p* =.102, ${\upeta }_{p}^{2}$= 0.004, nor on temporal location ratings, *F*(1, 728) = 2.53, *p* =.11, ${\upeta }_{p}^{2}$ = 0.003 (Table 4). Bayesian analysis (see Table 6) reinforced these nonsignificant findings, providing moderate evidence for the absence of contamination for both intensity ratings (BF_01_ = 3.46) and location ratings (BF_01_ = 3.18).

Regarding the effect of the temporal location factor on ratings of other characteristics, contrary to our prediction of no effect, the temporal location factor had a significant main effect on intensity ratings, *F*(2, 729) = 3.52, *p* =.03, ${\upeta }_{p}^{2}$ = 0.01 (Table 4). Unlike the prior two factors, this factor has three levels, so we conducted post hoc analysis. Post hoc analyses using Tukey's HSD correction showed that intensity ratings for stimuli presented at the start of the film were significantly lower than those presented at the end (diff =  − 5.62, 95% CI [− 10.22, − 1.02], *p* = 0.01). The Bayesian analysis yielded a BF_01_ = 2.21 (see Table 6), suggesting that while the ANOVA result was significant, the evidence in support of this effect was weak.

The temporal location factor also had a significant but small main effect on duration ratings, *F*(2, 728) = 3.09, *p* =.046), again with small effect size (${\upeta }_{p}^{2}$= 0.006). Post hoc analyses using Tukey's HSD correction did not reveal any significant differences between specific pairs of temporal locations: Middle–End (diff = 0.34, 95% CI [− 8.68, 9.36], *p* =.99), Start–End (diff = 8.14, 95% CI [− 0.68, 16.97], *p* = 0.08), and Start–Middle (diff = 7.80, 95% CI [− 0.86, 16.47], *p* = 0.09). Although there was a significant main effect of temporal location factor, the differences in duration ratings between locations were not large enough to produce significant *p* values. The Bayes factor also provided moderate evidence for the null effects (BF_01_ = 6.49), suggesting that temporal location factor effects on duration ratings were negligible.

Consistent with our prediction, no significant interaction effects involving unexpected effects were observed for any rating (*p* >.05). Bayesian analysis provided moderate-to-strong evidence for the absence of these interactions across all dimensions. Notably, the three-way interaction ($Intensity\times Duration\times Location$) yielded BF_01_ values ranging from 4.97 to 13.75, confirming that the ratings were robust against complex cross-characteristic contamination.

### Exploratory analyses

Although most participants accurately reported a single thunder event, some individuals did not perceive the event as intended, reporting either no thunder (14%) or multiple events (11%). To understand what may have happened in these instances, exploratory analyses were conducted to evaluate predictors of inaccurate perception. Participants reporting one instance (*n* = 741) were compared with those reporting no thunder (*n* = 133; see Supplemental Table 1 A) and, separately, with those reporting multiple instances (*n* = 111; see Supplemental Table 1B) using logistic regression analysis with demographic variables and stimulus characteristics (stimulus intensity, stimulus duration, stimulus temporal location) as predictors. The analysis comparing participants reporting no thunder versus one instance revealed that reporting error was significantly more likely for Black (OR = 0.39, *p* <.0001) and multiracial participants (OR = 0.50, *p* = 0.04) compared to White participants (reference group). Turning to the stimulus characteristics, reporting error was significantly less likely in the high-intensity (OR = 2.92, *p* <.0001) and long-duration (OR = 3.79, *p* <.0001) conditions, and more likely when the stimulus occurred at the start versus the end of the film (OR = 0.41, *p* = 0.0005). Errors were also more likely for participants with college education or higher (OR = 0.63, *p* = 0.02), those with higher income (marginally, OR = 0.66, *p* = 0.05), and Black participants (OR = 0.34, *p* <.0001) compared to respective reference groups. Again, high intensity (OR = 1.85, *p* = 0.003) and long duration (OR = 1.93, *p* = 0.002) were significantly associated with fewer errors. However, having the stimulus occur at the start versus the end (OR = 1.75, *p* = 0.02) was associated with lower odds of accurately perceiving a single stimulus event.

## Discussion

This study aimed to provide evidence regarding the validity of coverage ecological momentary assessment by simulating a situation wherein participants rated the characteristics of an experimentally manipulated stimulus after engaging with a 2-h video activity. The central question addressed was whether participants could accurately recall objective event characteristics given the potential for recall biases even over relatively short periods. The results offer insights into both the potential and the limitations of cEMA. Participants could distinguish and rate key characteristics of the stimulus event (intensity, duration, temporal location), suggesting cEMA holds promise for capturing information about events experienced over a 2-h period. However, recall was imperfect and reflected systematic inaccuracies and biases that were related to the objective stimulus characteristics. Regarding cross-characteristic influences, although we observed that responses could be statistically influenced by irrelevant stimulus characteristics, these effects were notably small. This suggests that while participants’ judgments are not entirely isolated from the broader stimulus context, the practical impact of this on data validity appears minimal. Our findings underscore the need for careful consideration when interpreting cEMA data, where those considerations are associated with the study objectives, and the findings suggest avenues for future research.

Participants clearly distinguished between high- and low-intensity sounds in their ratings, supporting the idea that cEMA can measure subjective experiences related to stimulus intensity. This is an important finding because it is likely that many cEMA studies will measure intensity of a phenomena of interest (e.g., pain, affective states, emotions). Nevertheless, although significant differences were detected, there was a lot of overlap presented between the intensity ratings of the two groups defined by low and high intensity, which was obvious in the density distributions of the scores by group. In considering this finding, we highlight the fact that this is a between-persons or cross-sectional examination of the data, wherein there is only a single intensity score for each participant. We also note that the response for intensity was 0 (no sound at all) to 100 (the loudest possible sound), which has no standardized external referent and is likely influenced by individual differences in peoples’ experience of sounds. Thus, these ratings can have considerable variability. This is a likely explanation, at least in part, for the overlap in the density distributions—an issue we have discussed in a recent paper (Stone et al., 2025). However, this issue is likely mitigated in typical cEMA protocols, which involve intensive repeated assessments of the same individual over time. By utilizing repeated measures, researchers can employ statistical techniques (such as person-mean centering or multilevel modeling) to isolate within-person variation from between-person differences. Thus, while the heterogeneity observed here highlights the challenges of comparing raw intensity scores across individuals, it poses less of a threat to the validity of associations detected in true within-person EMA designs.

We also found that higher objective intensity led to shorter duration ratings albeit with a very small effect size (η^2^ = 0.02), weakly suggesting potential cross-characteristic bias within cEMA ratings. This broadly aligns with Kahneman et al. (Kahneman et al., 1993) and Ariely et al.’s (Ariely, 1998) conclusion that recall, even over short periods, can be influenced by factors other than the specific dimension being queried. This finding goes to the point that some stimulus characteristics may be difficult to accurately measure in cEMA, although the bias does appear minor.

Results on duration ratings also offer a complex picture for cEMA applications. While participants' duration ratings were strongly associated with the objective duration (${\upeta }_{p}^{2}$= 0.17), the absolute agreement levels of durations were overestimated, particularly for the longer stimulus (mean differences of +  11 s and +  20 s for low and high duration conditions, respectively). Considering that only 31% of reports were within 5 s of the true value, these results suggest that obtaining precise duration estimates via 2-h cEMA may be challenging. Returning to the example mentioned for intensity, this error and the variability of the error as indicated by the density distributions could impact associations with passively measured outcomes. The findings also imply that duration data from cEMA might be better for relativistic comparisons (e.g., one event being longer than another) than for absolute accuracy, and researchers should be aware of a potential tendency towards overestimation in participant reports. Consistent with our second hypothesis, duration factor does not impact ratings of other characteristics.

Temporal location reporting had the strongest results with a large effect size (${\upeta }_{p}^{2}$ = 0.38), indicating participants discriminated well between events occurring at the start, middle, and end of the 2-h period. Overall accuracy was reasonable (approx. 70% within 15 min). cEMA might be reasonable for reconstructing the timing of salient discrete events throughout the reporting window. The result is tempered by the finding that there were also systematic biases in the ratings, with events occurring at the beginning being recalled later and events at the end being recalled occurring earlier than their actual time of occurrence. Moreover, the influence of temporal location on intensity ratings, with end events rated significantly higher than start events, was contrary to expectations but consistent with established memory research on recency effects (Ariely, 1998). Again, the effect size for this potential bias was small (${\upeta }_{p}^{2}$ = 0.01). This observation may reflect a recency bias, where the most recently experienced stimulus is more available or salient in memory, thereby influencing recall of its characteristics. While a significant main effect of temporal location on duration ratings was also found, the effect size was negligible (${\upeta }_{p}^{2}$ = 0.006), and post hoc tests comparing specific locations were nonsignificant, indicating this cross-characteristic influence is weak or unreliable in this context.

Moving to the exploratory analyses, an important finding for interpreting these results is that about a quarter of the participants did not accurately report the occurrence of a single stimulus event—either missing it entirely or reporting multiple occurrences. Our analyses provided some clues for understanding the phenomenon. As one might expect, the intensity of stimuli was a strong predictor of whether the event was perceived at all, where most of the absence of reporting was of low-intensity stimuli. One conclusion may be that less intense or salient events may be underdetected using cEMA. An alternative explanation is that the finding was a failure of our design to present a stimulus that was discriminable from the background video. That is, it was simply too weak. Arguing against this interpretation was the successful pilot testing of the stimuli. Yet there may have been more background noise for participants using the protocol in the field that we did not anticipate. Objective stimulus duration also strongly predicted correct event perception, also indicating that shorter stimuli were less likely to be accurately recalled. This underscores a fundamental challenge for cEMA: understanding whether a targeted event occurrence is sufficiently distinctive (or attended to by the participant) for it to be accurately recalled. The fact that correct perception of the event was strongly predicted by stimulus intensity and duration suggests cEMA may be less reliable for detecting subtle, brief, or low-salience events. The exploratory findings associating race and education/income to perception accuracy also suggest participant factors may interact with event characteristics in influencing data quality, though these require cautious interpretation and further study.

The insights gained must be interpreted in light of the study's strengths and limitations. Strengths of this study include the large sample, experimental control over stimulus characteristics allowing causal inference, simulation of a relevant 2-h EMA recall interval, and inclusion of attention checks. However, the simulated environment in which the stimulus event was experienced limits direct generalization to the free-living settings usual for EMA studies, where context is more variable, and recall occurs amidst daily life demands. This study’s findings are also limited by the use of a single, discrete auditory stimulus, and we have no evidence that it generalizes to the more complex constructs being studied in EMA. Another limitation is the exclusion of participants who misreported the number of events, resulting in our primary findings on rating accuracy pertaining only to those who successfully detected the single event. An implication of this concern is that we may have overestimated the effect of these characteristics compared to the full population experience. Finally, the fixed 2-h interval is only one choice of many that are available to cEMA users, and we do not know if these results generalize to the accuracy of cEMA over shorter or longer periods.

Despite these limitations, this study provides new data that is pertinent to the adoption of the cEMA technique. They suggest that while 2-h recall is feasible for capturing basic event features—showing sensitivity to objective characteristics as hypothesized—the assumption of independent and unbiased recall is not fully supported. We observed systematic biases (duration overestimation, temporal compression) and cross-characteristic influences (intensity affecting duration ratings and location affecting intensity ratings), although the small effect sizes associated with these effects suggest that they may have limited practical impact. More broadly, the finding that event detection itself was imperfect highlights the need to carefully consider the nature of events targeted by cEMA—very brief or low-intensity events may be particularly prone to being missed. Researchers employing cEMA should consider these potential biases during study design and data interpretation. For instance, analyzing relative changes within individuals might be more robust than relying on the absolute accuracy of single reports, especially for duration. Future research is recommended to replicate and extend these findings. Studies could investigate recall accuracy in true real-life settings, examine different types of events (e.g., social interactions, symptom episodes) and modalities, explore varying coverage window lengths, and explicitly test strategies to potentially mitigate observed biases (e.g., providing anchors, using different question formats). Further investigation into the demographic factors associated with perception accuracy is also warranted to understand potential equity issues in EMA data quality.

## Conclusion

In conclusion, this study provides new evidence about the use of cEMA for recalling events over a 2-h period based on experimental manipulation of the to-be-recalled stimulus. While participants demonstrated the ability to discriminate the objective intensity, duration, and temporal location of the auditory stimulus—supporting the ability of this method for capturing event characteristics—this capability must be considered alongside the study limitations. We observed small to modest systematic biases, including overestimation of duration, biases in temporal location, and potential for cross-characteristic influences. In conducting cEMA work, researchers should consider challenges in basic event detection, particularly for less salient stimuli. Therefore, while offering considerable optimism about the cEMA procedure, these results emphasize the need for researchers to carefully consider potential inaccuracies and context-dependent biases inherent in cEMA data when designing studies and interpreting findings.

## Data Availability

The study materials and data used in the current study are available at [https://osf.io/xfwtn/files/osfstorage]. None of the reported studies were preregistered.

## Footnotes

1 Ecological momentary assessment (EMA) is also referred to by other terms, including experience sampling method (ESM) and ambulatory assessment.

## Code availability

The analytic code used in the current study is available at [https://osf.io/xfwtn/files/osfstorage].
